# Pulmonary emphysema is a predictor of pneumothorax after CT-guided transthoracic pulmonary biopsies of pulmonary nodules

**DOI:** 10.1371/journal.pone.0178078

**Published:** 2017-06-02

**Authors:** Derik Lendeckel, Marie-Luise Kromrey, Till Ittermann, Sophia Schäfer, Birger Mensel, Jens-Peter Kühn

**Affiliations:** 1 Institute of Diagnostic Radiology and Neuroradiology, University Medicine Greifswald, Greifswald, Germany; 2 Institute of Community Medicine, University Medicine Greifswald, Greifswald, Germany; 3 Department of Radiology, Universitätsklinikum Dresden, Carl Gustav Carus University Dresden, Dresden, Germany; National and Kapodistrian University of Athens, SWITZERLAND

## Abstract

**Purpose:**

Pneumothoraces are the most frequently occurring complications of CT-guided percutaneous transthoracic pulmonary biopsies (PTPB). The aim of this study was to evaluate the influence of pre-diagnostic lung emphysema on the incidence and extent of pneumothoraces and to establish a risk stratification for the evaluation of the pre-procedure complication probability.

**Material and methods:**

CT-guided PTPB of 100 pre-selected patients (mean age 67.1±12.8 years) were retrospectively enrolled from a single center database of 235 PTPB performed between 2012–2014. Patients were grouped according to pneumothorax appearance directly after PTPB (group I: without pneumothorax, n = 50; group II: with pneumothorax, n = 50). Group II was further divided according to post-interventional treatment (group IIa: chest tube placement, n = 24; group IIb: conservative therapy, n = 26). For each patient pre-diagnostic percentage of emphysema was quantified using CT density analysis. Emphysema stages were compared between groups using bivariate analyses and multinomial logistic regression analyses.

**Results:**

Emphysema percentage was significantly associated with the occurrence of post-interventional pneumothorax (p = 0.006). Adjusted for potential confounders (age, gender, lesion size and length of interventional pathway) the study yielded an OR of 1.07 (p = 0.042). Absolute risk of pneumothorax increased from 43.4% at an emphysema rate of 5% to 73.8% at 25%. No differences could be seen in patients with pneumothorax between percentage of emphysema and mode of therapy (p = 0.721).

**Conclusion:**

The rate of lung emphysema is proportionally related to the incidence of pneumothorax after CT-guided PTPB and allows pre-interventional risk stratification. There is no association between stage of emphysema and post-interventional requirement of chest tube placement.

## Introduction

Computed tomography (CT)-guided percutaneous transthoracic pulmonary biopsy (PTPB) of suspect pulmonary nodules is an important element in the diagnostic routine. It is essential for obtaining tissue samples to distinguish and classify tumor entities. CT-guided PTPB is known to be less risk entailing for the patient compared to surgical procedures, as it is less invasive and still provides a high diagnostic accuracy. The most frequent complication after PTPB is the occurrence of a pneumothorax, which has an incidence of up to 50–60% after PTPB [1–6] and can lead to a considerable reduction in the health state of the patient and longer hospitalization [7].

To minimize the rate of complications, the prior aim of recent research is to identify predictors for the occurrence of pneumothoraces. Interestingly, authors revealed contrary results, which reflect the complicity of the topic. For example, a recently published review reported a concurrence of various factors such as lesion size, lesion depth and the presence of emphysema to determine the incidence of post-procedure pneumothoraces [8]. In contrast to that, a retrospective analysis showed the body position as the main predictor with no influence of lesion size and depth among others [1]. Laurent et al. suggest lesion size, location, depth and difficulty of the procedure as significantly influencing the rate of pneumothoraces [9].

Especially pulmonary emphysema is a controversially discussed predictor for the occurrence of a pneumothorax associated with transthoracic biopsies. Results of recently published studies showed no influence of emphysema on the incidence of pneumothoraces at all [1] or reported the emphysema to affect the extent of pneumothoraces and the need of chest tube placement, but not the incidence per se [9]. Cox et al. suggest a synchronicity of lesion size and emphysema as predictors [10]. A recent publication by Chami H. et al. deals with the association between the emphysema percentage quantified by CT volumetric lung analysis and the rate of pneumothorax after PTPB [11]. They showed pulmonary emphysema to be an independent predictor of pneumothorax. The rate of chest tube placement was not related to the percentage of emphysema. The authors propose, that pre-interventional assessment of pulmonary emphysema by a quantitative analysis could help biopsy planning. However, a risk stratification of pulmonary emphysema for the occurrence of pneumothorax was not performed [11].

Therefore, the purpose of our study was to investigate if emphysema was a possible predictor for the occurrence of pneumothorax after CT-guided PTPB and to establish a risk stratification of pulmonary emphysema and pneumothorax rate in order to optimize pre-interventional planning.

## Materials and methods

This retrospective study was approved by the Institutional Review Board of the University Medicine of Greifswald, Germany. Informed consent was waived and patient’s data were assessed anonymously.

### Study population

Study subjects were recruited from a patient database of performed CT-guided transthoracic biopsies. The interventions were performed between January 2012 and October 2014 following a standardized procedure using a cutting needle system without coaxial technique. 235 CT-guided PTPB conducted in this period, were reviewed and classified into two groups according to the presence of post-interventional pneumothorax. 50 patients from each group (group I: no pneumothorax; group II: pneumothorax) were randomly selected and enrolled into the conducted study. The mean age of the whole study cohort was 67.05 ± 12.82 years (age range 7–86 years) with 73 male and 27 female patients. Needle sizes encompassed 14G (n = 3, 3%), 16G (n = 10, 10.1%) and 18G (n = 86, 86.9%). In one person, needle size was not documented.

Group I included subjects with absence of pneumothoraces after PTPB and group II patients with occurrence of pneumothorax. Group I held 35 male and 15 female patients with a mean age of 65.98 years ± 12.71 years standard deviation (SD). Within group I 2 subjects were punctuated with a 14G needle size (4%), 8 with 16G (16%) and 40 with 18G (80%). Group II consisted of 38 males and 12 females with a mean age of 68.12 ± 12.98 years. In group II 14G needles were used once (2%), 16G twice (4.1%) and 18G 46 times (93.9%).

For each group, biopsies were analyzed with regard to the stage of pulmonary emphysema, age, gender, lesion size and length of interventional pathway. Group II (pneumothorax group) was furthermore divided into group IIa containing 24 PTPB with the need of chest tube placement and group IIb containing 26 biopsies with conservative therapy approach.

### Quantitative assessment of pulmonary emphysema

Selected patients had a complete lung CT within 60 days before the procedure. Quantitative assessment of pulmonary emphysema was performed using the Pulmo 3D Workspace of the Syngo.Via software (Siemens Healthcare, v1.1.0.18). Validation of this software for quantification of pulmonary emphysema stages has been proven in previously accomplished studies [12, 13]. The software was used to perform automatic segmentation of lung parenchyma and calculate the total lung volume, as well as percentage of emphysema for each lung compared to total lung volume. Emphysema was defined as tissue attenuation lower than -950 Hounsfield Units (HU) inside the lung boundaries, as this cut-off is approved to provide the best correlation to microscopic quantification [14, 15].

### Evaluation of pneumothorax

**Fig 1:**
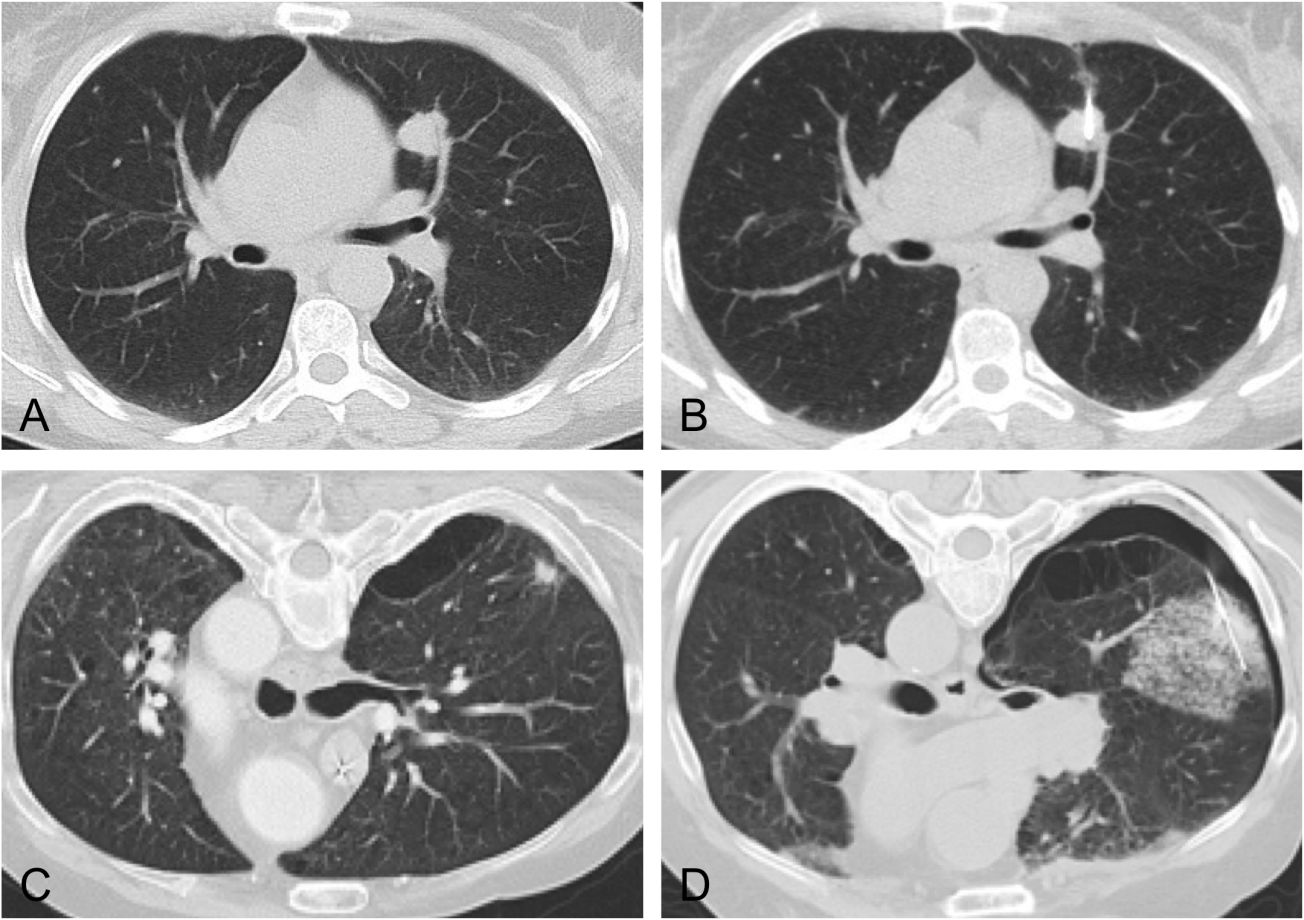
Exemplary images of CT-guided percutaneous transthoracic pulmonary biopsies with and without lung emphysema. Pulmonary nodule in otherwise normal lung parenchyma before (A) and after PTPB (B). No post-interventional complication was detected. Pictures (C) and (D) show a patient with pulmonary emphysema. After PTBP (D) CT examination revealed pneumothorax and parenchyma hematoma.

The presence of pneumothoraces was confirmed with the help of post-procedure CT-imaging performed directly after intervention. Fig 1 shows the image of a CT-guided PTPB with and without emphysema and consecutive pneumothorax. Analyses of the extent of pneumothoraces were accomplished by radiologists, who performed the interventions. It was within discretion of the interventional radiologists to decide if post-interventional pneumothoraces needed further treatment such as chest tube placement. The estimated cut-off for the decision for chest tube therapy was a pneumothorax size of more than 2.5 cm or a considerable lapse of the patient's condition.

### Further potential predictors

Demographic patient data like age and gender were extracted from the in-house medical database. In addition, procedure-linked data like lesion size, needle size, length of interventional pathway and number of pleura passes were collected by reviewing the images and reports of the corresponding CT-guided PTPB using the image processing software IMPAX (PACS, Agfa HealthCare N.V., v6.5.2.114).

### Statistical analyses

Continuous data are described as mean and standard deviation, dichotomous data as absolute numbers stratified by groups with and without pneumothorax. The association between emphysema rate and pneumothorax was tested by logistic regression adjusted for age, sex, lesion diameter and length of interventional pathway for the yes/no outcome and by multinomial logistic regression for the outcomes pneumothorax occurrence with drainage, pneumothorax without drainage and no pneumothorax. A p < 0.050 was considered as statistically significant. Data analyses were conducted by Stata 14.1 (Stata Corporation, College Station, TX, USA).

## Results

**Fig 2:**
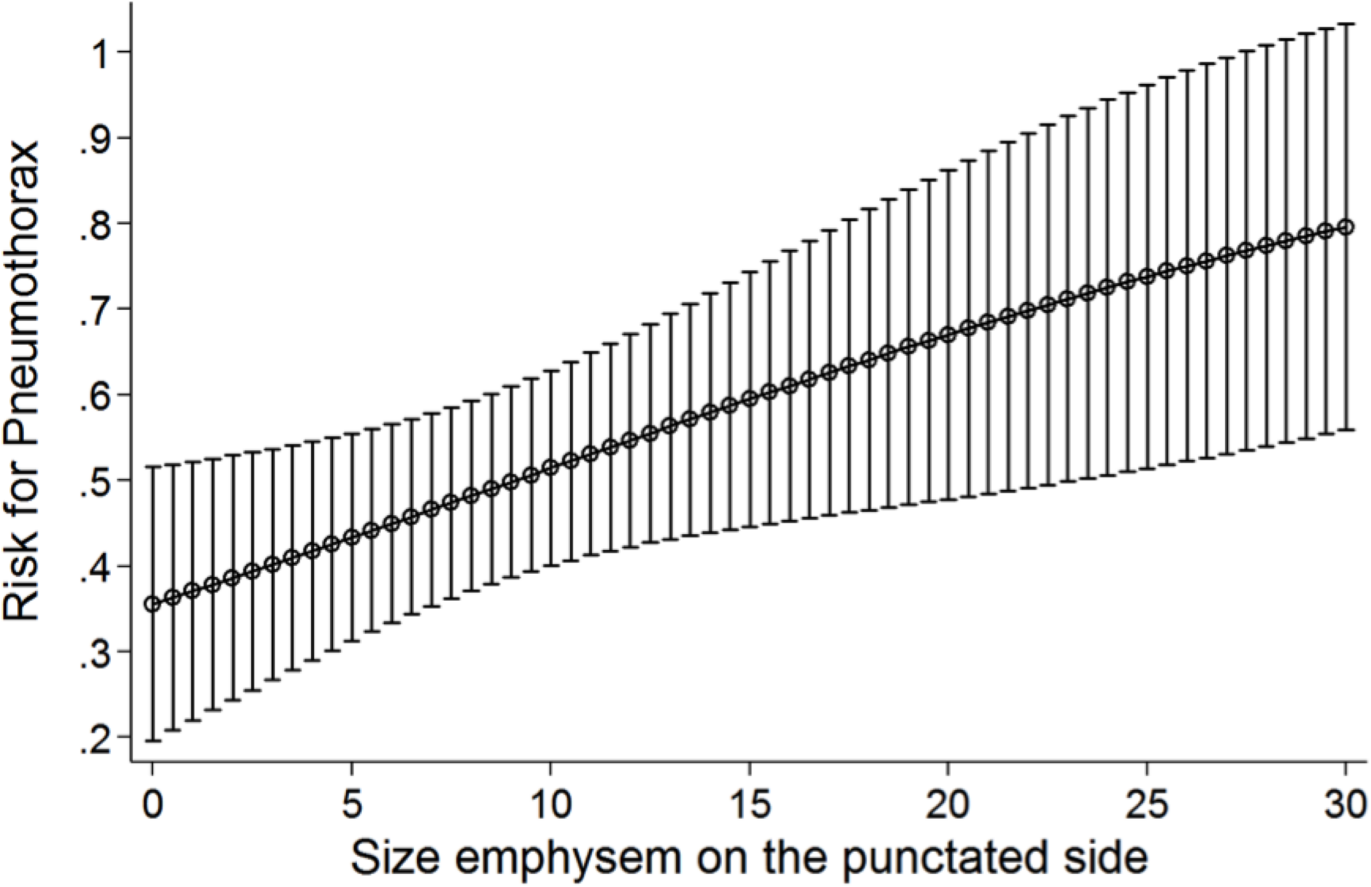
Risk of pneumothorax according to the stage of pulmonary emphysema. Multinomial logistic regression shows linear correlation of increasing emphysema percentage and a heightened risk of the incidence of pneumothorax after CT-guided PTPB.

In group I and II means and standard deviations of the parameters stage of pulmonary emphysema, age, gender, lesion size and length of interventional pathway were analyzed and are displayed in Table 1. The overall mean percentage of emphysema after lung biopsy in our study population was 8.6 ± 7.6%. Statistical analyses revealed a significantly higher percentage of emphysema in the group with post-interventional pneumothorax (group II, mean 10.4 ± 7.5%) compared to the group without (group I, mean 6.7 ± 7.2%) (p = 0.006). Adjusted for age, sex, lesion diameter and length of interventional pathway this ensued an odds ratio (OR) of 1.07 with a 95%-confidence interval (CI) of 1.01–1.14 and a p-value of 0.042 –the risk to develop a pneumothorax after CT-guided biopsy, therefore, was 7% per percent emphysema. For example, analyses of group I and II revealed an approximate pneumothorax risk of 43.4% after CT-guided PTPB at an emphysema percentage of 5% increasing to 73.8% with an emphysema percentage of 25% (Fig 2).

**Table 1:** Means and standard deviations of analyzed parameters.

parameter	group I	group II		overall
		IIa	IIb	
**age** in years (±SD)	65.98 (±12.71)	68.12 (±12.98)		67.05 (±12.82)
		69.63 (±9.64)	66.73 (±15.50)	
**gender**	35m/15f	38m/12f		73m/27f
		21m/3f	17m/9f	
**emphysema** (±SD)	6.68% (±7.22)	10.55% (±7.46)		8,61% (±7.6)
		10.40% (±7.48)	10.69 (±7.58)	
**lesion size** in cm **(**±SD)	4.45 (±2.43)	2.84 (±2.06)		3.65 (±2.38)
		3.08 (±2.65)	2.62 (±1.32)	
**length of pathway** in cm **(**±SD)	7.90 (±2.34)	8.90 (±3.23)		8.40 (±2.85)
		8.50 (±2.67)	9.28 (±3.69)	
**pleura passes (**±SD)	1.66 (±0.69)	1.36 (±0.48)		1.51 (±0.61)
		1.29 (±0.46)	1.42 (±0.50)	

There was no significant influence of demographic patient parameters such as age (OR: 1.01; 95% CI: 0.97–1.05; p = 0.617) and gender (OR: 0.65; 95% CI: 0.23–1.87; p = 0.425) on the occurrence of pneumothorax. Lesion size was confirmed as strong predictor for pneumothorax after CT-guided PTPB (OR: 0.71; 95% CI: 0.57–0.90; p = 0.004). In contrast, the length of interventional pathway was not associated with post-interventional pneumothorax (OR: 1.12; 95% CI: 0.95–1.33; p = 0.161), as was not the number of pleura passes (OR: 0.51; 95% CI: 0.20–1.31; p = 0.164). Likewise, needle size was not significantly associated with development of pneumothorax (p = 0.114).

Regarding the subgroup analysis after occurrence of pneumothorax–chest tube placement (group IIa) versus conservative treatment (group IIb)–, emphysema had no influence on the extent of the pneumothoraces and the necessary therapy: No significant difference could be shown between group IIa (10.4 ± 7.5%) and group IIb (10.7 ± 7.6%) (OR: 0.98; 95% CI: 0.90–1.07; p = 0.721). Furthermore, lesion size (OR: 1.21; 95% CI: 0.84–1.74; p = 0.303) or needle size (p = 0.613) were not found to be predictors for the need of chest tube placement.

## Discussion

This study investigated the association between pneumothorax after CT-guided percutaneous transthoracic pulmonary biopsies (PTPB) and the severity of pulmonary emphysema. Pulmonary emphysema was found to be an independent predictor of post-interventional pneumothorax. Interestingly, pulmonary emphysema was not associated with the severity of pneumothorax (e.g. requirement of post-interventional chest tube placement). The data presented in this study may be used by interventional radiologists to estimate the risk of iatrogenic pneumothorax before CT-guided PTPB, which could be important for pre-interventional planning.

Pneumothorax is the most frequent complication of CT-guided PTPB with a reported post-procedure incidence ranging between 17–60% [6, 16, 17]. A chest tube placement for pneumothorax therapy is needed in 1%-14% of cases [16–18]. Although several studies supposed lung emphysema as an important risk factor for post-interventional pneumothoraces [8, 10, 11], a concluding consensus has not yet been established, as there are diverse results in recent literature revealing no correlation [1]. The findings of this study are yet another strong indicator for the essential role of emphysema on the occurrence of post-PTPB pneumothorax, as emphysema was, besides the lesion size observed, the only predictor for the incidence of pneumothoraces after intervention.

Furthermore, our results indicate, that emphysema of the lung has no influence on the need of chest tube placement as a parameter for the extent of pneumothoraces. The recent literature is ambivalent regarding the role of emphysema determining pneumothorax therapy options. Laurent et al., for example, reported a correlation between severe emphysema and the need for chest tube placement [9]. In contrast, Chami et al. did not find any relationship between chest tube therapy and percentage emphysema [11].

The diverse findings of these studies could be a result of the different approaches to measure lung emphysema. Several studies were limited to visual evaluation of chest CTs to estimate the percentage emphysema before CT-guided PTPB [8, 10, 19]. Chami et al. were the first to propose an automated quantification of emphysema percentage by using an analyzing software [11]. The reliability of these automated quantification approach is pointed out by this study, as it fully confirms the results of the referred study with the help of another analyzing software algorithm and a different study design. In contrast to the approach of Chami et al., this study preselected the CT-guided PTPB according to their outcome and performed retrospective analyzes of the emphysema percentage in each group.

To evaluate the feasibility of a CT-guided PTPB, the risk to induce a severe complication should be put into consideration as well as the patient's general condition and the probability to successfully hit the lesion with the biopsy needle and obtain representative material [20]. A likelihood assessment of post-procedure pneumothoraces is therefore an essential part of pre-interventional planning, as their occurrence could lead to a considerable worsening of the patient's state and prolong the duration and costs of hospitalization. The interventionalist’s possibility to assess the probability of post-procedure pneumothoraces is yet limited to his own evaluation based on experience and subjective criteria like the patient's general condition and the presence of possible predictors.

Therefore, an automated quantification of the lung emphysema percentage could be an important approach in clinical routine to reliably and objectively predict the risk of a pneumothorax after CT-guided transthoracic pulmonary biopsies. Chami et al. already showed the capability of automated quantification analysis to evaluate the predictors of post-procedure pneumothoraces, but they did not establish an adequate pre-interventional risk stratification [11]. Our study revealed an increase in the risk of pneumothorax development after PTPB of 7% per percent emphysema–the pneumothorax rate increased from 43.4% for an emphysema rate of 5% to 73.8% for an emphysema rate of 25%. The same analyses should be performed for the influence of lesion size on the post-procedure pneumothorax rate, as this and previous studies indicated it as a possible additional major predictor.

There exist several factors possibly influencing the post-interventional pneumothorax rate. In our study, lesion size was strongly correlated with pneumothorax occurrence, whereas other the other risk factors assessed–age, gender, needle size, length of interventional pathway and number of pleura passes–did not show a significant correlation. In order to determine risk stratification of emphysema, data were adjusted for lesion diameter. However, regarding current literature, there exist other factors with an impact on PTPB, such as incomplete pulmonary fissures or previous radiation or chemotherapy [21], which were not examined in our study.

There are several limitations to our study. First, there is only a restricted number of subjects included. Furthermore, other potential predictors for PTPB examined in previous studies, such as pulmonary fissures, radiation or chemotherapy, diffuse lung disease or previous pneumothorax [21], were not investigated. In addition, the study results may be influenced by other potential, hitherto unknown confounders, such as the interventional radiologist as confounder his- or herself.

In conclusion, the rate of lung emphysema is proportionally related to the incidence of pneumothorax after CT-guided PTPB. There is no association between stage of emphysema and the need for chest tube placement. The data presented in this study allow a pre-interventional risk stratification, which could be important to assess the feasibility and verify the indication of the intervention. The authors suggest the routinely use of automated quantification of the emphysema percentage in clinical settings. For further research, the aim should be the establishment of a risk stratification for all possible predictors, in particular lesion size.
